# Assessing Myocardial Perfusion after Myocardial Infarction

**DOI:** 10.1371/journal.pmed.0030131

**Published:** 2006-03-28

**Authors:** Houman Ashrafian, Girish Dwivedi, Roxy Senior

## Abstract

Ashrafian and colleagues describe the use of myocardial contrast echocardiography to assess a 63-year-old man with ischemic heart disease.

## DESCRIPTION of CASE

A 63-year-old man presented to our accident and emergency department with a 2-hour history of crushing central chest pain, excessive sweating, and difficulty in breathing. Past history included non-insulin-dependent diabetes mellitus controlled by oral hypoglycaemic agents.

**Figure 1:**
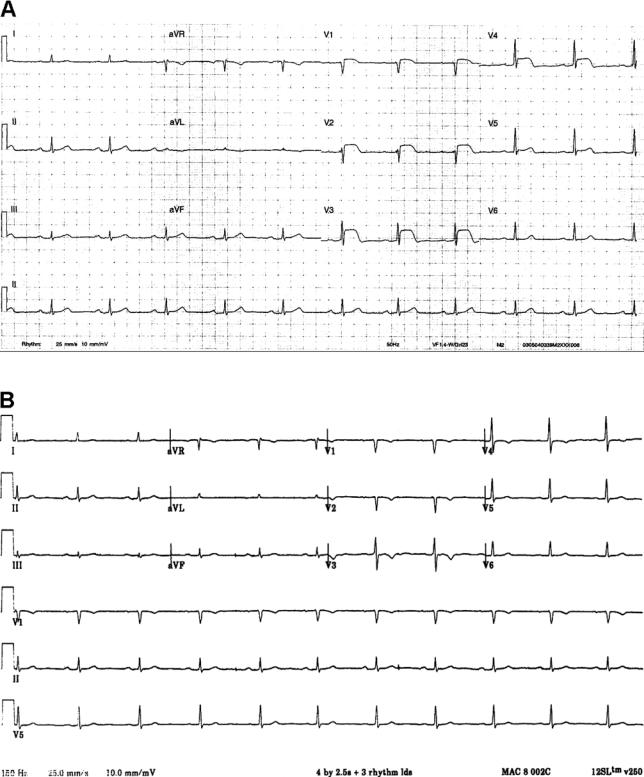
Electrocardiogram on Admission and after Thrombolytic Therapy (A) Electrocardiogram on admission showing >2 mm ST elevation in chest leads V1–V4. (B) Electrocardiogram after thrombolytic therapy showing subsidence of elevated ST segments.

On examination, he appeared unwell, with pulse of 94 beats per minute, blood pressure of 124/68 mm Hg, and respiratory rate of 20 breaths per minute. Jugular venous pressure was not elevated, but chest auscultation revealed bilateral basal crackles. Cardiovascular system examination was unremarkable. His electrocardiogram on admission showed >2 mm ST segment elevation in chest leads V1–V4 ( Figure 1A).

### What Was the Clinical Diagnosis and Treatment?

The combination of typical chest pain and electrocardiographic changes were characteristic of acute anteroseptal myocardial infarction. A presumptive diagnosis of probable left anterior descending (LAD) coronary artery occlusion was made. Our patient was promptly thrombolysed with recombinant plasminogen activator and managed as per our hospital's acute myocardial infarction (AMI) protocol. This involved administration of diamorphine, aspirin, intravenous nitrates, heparin, a beta-blocker, an angiotensin converting enzyme inhibitor, and a statin.

Within 30 minutes of thrombolytic therapy, there was complete cessation of pain and subsidence of elevated ST segments ( Figure 1B). His peak CK and CK-MB were elevated at 577 IU and 68 IU, respectively. Wall motion abnormalities on transthoracic echocardiogram were identified in the resting LAD territory. His further stay in the coronary care unit was uneventful.

### What Was the Subsequent Differential Diagnosis?

Our patient presented with electrocardiogram changes of a myocardial infarction (MI). Although this is most likely due to coronary occlusion, the ST elevation may occur for reasons other than infarction ( Box 1). Moreover, infarction need not result from coronary occlusion ( Box 2). The electrocardiogram and echocardiogram thus both suggested a high probability of ischemia in the LAD territory. In order to stratify our patient's prognosis, it would be important to determine whether the patient did indeed have the implied single vessel (LAD) coronary artery disease and whether the infarct territory was optimally revascularised at macro- and microvascular levels. As part of post-MI risk stratification, our patient underwent vasodilator stress radionuclide perfusion and myocardial contrast echocardiography (MCE).

**Figure 2:**
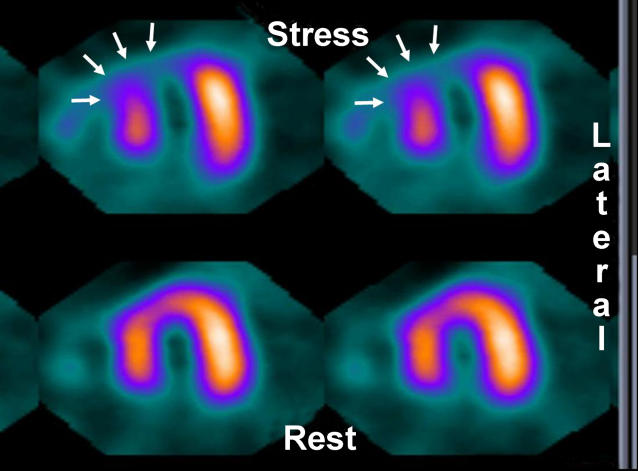
Radionuclide Imaging Showing a Reversible Perfusion Defect in the Septum The defect is shown by decreased tracer concentration (white arrows) in the whole of the septum after stress (seen in the short axis view).

Radionuclide imaging ( Figure 2) showed a reversible perfusion defect in the septum as shown by decreased tracer concentration after stress in the whole of the septum (seen in the short axis view). This defect indicated flow-limiting coronary artery disease in the LAD territory as suspected from the preliminary clinical assessment.

**Figure 3:**
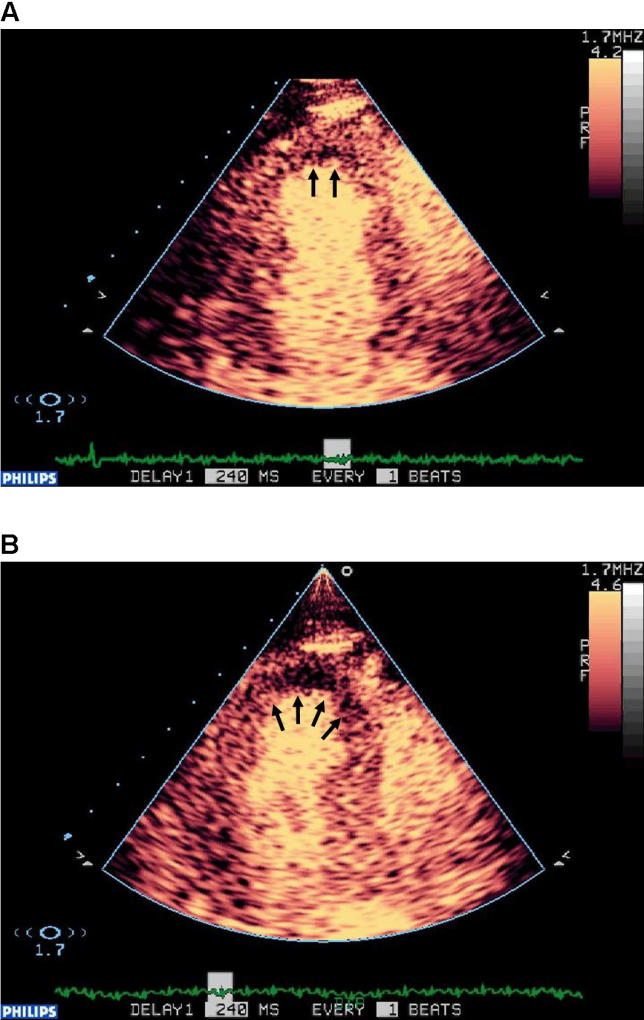
MCE: Apical Three-Chamber View Assessment (A) Apical three-chamber view assessment at rest. This assessment demonstrates a correspondingly reduced contrast uptake in the apical sub-endocardium representing an infarcted region (two black arrows) whilst the remaining myocardium shows normal opacification. (B) Apical three-chamber view assessment following stress. This assessment demonstrates markedly reduced contrast uptake in the entire septum (black arrows) suggesting mid-LAD disease.

MCE is shown in Figure 3. In Figure 3A, an apical three-chamber view assessment at rest demonstrates a correspondingly reduced contrast uptake in the apical sub-endocardium representing an infarcted region, whilst the remaining myocardium shows normal opacification. In Figure 3B, an apical three-chamber view assessment following stress demonstrates markedly reduced contrast uptake in the entire septum, suggesting mid-LAD disease.

### What Was the Final Diagnosis and Treatment?

**Figure 4:**
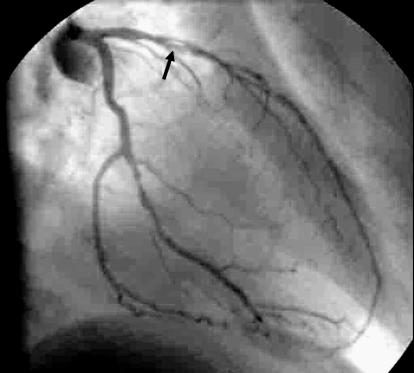
Coronary Angiography Showing Flow-Limiting Coronary Stenosis in the Mid-LAD Flow-limiting coronary stenosis is designated by the black arrow.

The patient underwent coronary angiography, which showed flow-limiting coronary stenosis in the mid-LAD ( Figure 4 and Video S1). This was successfully treated with coronary angioplasty with stent implantation before discharge from hospital.

## DISCUSSION

Major advances have been made in reperfusion therapy for AMI. Pharmacological developments have led to the introduction of recombinant thrombolytic therapies, which are increasingly safe and effective [ 1]. Moreover, the widespread application of catheter-based therapy, be this in the guise of primary or facilitated angioplasty, have dramatically delivered on the promise of reduced short-term and long-term AMI-related mortality [ 2]. This success derives from the expeditious salvage of vulnerable but viable myocytes threatened by occlusion of upstream vasculature by the clearance of flow-limiting arterial disease. Such salvage translates into retained myocardial function and increased chances of survival [ 3].

### Assessing the Success of Thrombolysis

However, pharmacological or mechanical reperfusions are not without risk and financial burden, and should be preferentially directed to a viable myocardium [ 4]. Effective reperfusion should therefore aim to reinstate the macro- and microvascular supply to only living myocytes. Ideally, a patient undergoing emergent thrombolytic therapy should be evaluated to see whether thrombolysis was successful. If it was unsuccessful, the patient should be immediately referred for rescue angioplasty.

Even if thrombolysis appears to be clinically successful, as in our patient, before more elective interventional therapy is considered it is prudent to quantify what proportion of dysfunctional myocardium may be viable and hence salvageable. This proportion is a marker of a patient's prognosis. Moreover, even if angioplasty—a therapy that is fast becoming the standard of care worldwide—rather than thrombolysis is the primary therapy for the AMI, assessing myocardial perfusion or lack thereof still informs prognosis. (In most cases an epicardial coronary vessel recanalised by angioplasty should establish flow at the microvascular coronary level; this may not happen in all cases and flow to the infarct myocardium may be diminished despite the angiographic documentation of reflow in the infarct-related artery; this is termed “no-reflow.”) [ 5]

How would these patients be stratified? Although clinical features such as the relief of pain and the absence of electrocardiographic Q waves have widely been regarded to imply a good response to thrombolysis and hence a residual mass of viable myocardium, they have been shown to be imprecise [ 6]. More recently, other imaging modalities have been advocated for the evaluation of myocardial viability, including radionuclide techniques, dobutamine echocardiography, magnetic resonance, and positron emission tomography [ 7].

### A New Technique for Assessing Myocardial Viability

MCE has emerged as one of the most effective of these modalities and has the added benefit of being available immediately at the patient's bedside; broadly, this technique relies on the application of echocardiographic ultrasound waves to intravascular microbubble contrast media that reflect these signals (echoes) [ 8, 9]. Microbubbles are stable bubbles of gas encased in polymeric shells. By careful design of the shells and the microbubbles, strong echoes are produced by the acoustic impedance mismatch. Furthermore, serendipitously, the size and constitution of the bubbles ensures that they coincidentally resonate within the frequency of normal cardiac ultrasound; they thus further improve signal-to-noise ratio. They are confined solely to the vasculature, and hence their presence and density is a surrogate for blood capillary volume. Resting contrast absence implies a non-viable myocardium, since these segments have chronically been starved of blood-delivered nutrients. Conversely, the presence of contrast implies a potentially viable myocardium as these regions are perfused. Finally, stress-induced contrast deficiencies imply a myocardium that is vulnerable due to vascular compromise.

How does MCE compare with other imaging modalities? Technetium-99m Sestamibi single-photon emission computed tomography is the most commonly used clinical imaging modality assessing myocardial perfusion; it has been successful in predicting coronary stenosis. A number of studies have compared these two modalities in the context of coronary artery disease and have broadly shown that MCE was equal or superior to single-photon emission computed tomography during dipyridamole stress for the diagnosis of coronary artery disease [ 10]; MCE does so at the bedside without the concomitant radiation risks.

In the specific setting of MI, by demonstrating an area of impaired perfusion (through microbubble paucity) MCE can define the vulnerable myocyte territory and make the diagnosis of MI efficiently [ 11]. In doing so, it can also specify the vessel that subtends this territory and is compromised by vessel occlusion. Once an intervention is performed, contrast studies can demonstrate the reversal of microbubble paucity and therefore adjudicate the success of revascularisation [ 12]. Importantly, as the most compelling prognostic factors after MI are the extent of myocyte loss, myocardial remodelling, and the resulting myocardial dysfunction, by quantifying the residual non-perfused myocardium an accurate estimate of dysfunctional myocardium can be made with immediate prognostic value. MCE can therefore predict the extent of residual myocardial viability and the degree of LV remodelling after MI [ 13]. It is important to note that in patients with a medium probability of coronary artery disease and no prior MI, MCE was at least as effective as the widely used radionuclide imaging [ 14]. Moreover, MCE correlates well with radionuclide imaging in patients with AMI post-revascularisation [ 15].

Indicating the value of MCE, in a recent study of >1,000 patients with chest pain presenting to an emergency department, MCE was found to be the best predictor of both short-term (48 hours) and long-term (up to 2 years) outcome (death, MI, revascularisation, unstable angina, and congestive heart failure). Unlike data collected from other modalities, MCE data were available immediately at the bedside after hospital presentation, and they remained superior even after all clinical, electrocardiographic, and serum biomarker information were taken into account [ 16].

### Limitations

While the literature strongly advocates the use of perfusion imaging to assess the presence of coronary artery disease and to direct further therapy, perfusion imaging in general and MCE in particular do have some limitations. The first and most important limitation is that of training; clearly, the application of this novel technique relies on specialist expertise. In order for “real-world” studies to reflect existing studies, a significant learning curve will have to be traversed. Secondly, while readily available, at present no microbubble contrast agents have been licensed for clinical practice. Although it is likely that they will be licensed, it is important to complete large-scale studies for regulatory bodies to be satisfied that these agents are free of deleterious bioeffects. To date, such studies have confirmed the safety and utility of these agents. Ultimately, the clinical application of MCE must be tempered with the knowledge that the capacity of perfusion analysis is limited in myocardial segments such as the baso-lateral walls that are prone to greater errors.

### Conclusion

MCE is a rapid bedside method for assessing myocardial perfusion. Among other cardiovascular conditions, and as with other forms of perfusion imaging, MCE in MI provides significant diagnostic information both in the acute sense and with regard to revascularisation strategy. Ultimately, these microbubbles can also be generated carrying therapeutic agents or carrying receptors or ligands within their membranes. They may, therefore, provide a means to direct intravascular treatments specifically to the site of active disease, as envisaged by Ehrlich when he described his magic bullet therapy [ 17].

## Supporting Information

Video S1 Coronary Angiography Showing Flow-Limiting Coronary Stenosis in the Mid-LAD (463 KB AVI)

**Box 1. Causes of ST-Elevation in the Absence of MI**

Normal variant; often an ethnic variant in African-American males
Left ventricular hypertrophy
Pericarditis
Hyperkalaemia
Brugada syndrome
Periresuscitation
Neurological events (e.g., seizure, subarachnoid haemorrhage)
Post-cardioversion
Pulmonary embolism

**Box 2. Causes of ST-Elevation MI in the Absence of Coronary Disease**

Coronary thrombosis, due to coagulopathy (e.g., homocysteinaemia, protein C/S deficiency, and use of oral contraceptives)
Vasculitis (e.g., Kawasaki syndrome and collagen disorders)
Cocaine
Coronary spasm
Embolic events (e.g., paradoxical emboli from patent foramen ovale or endocarditis)
Congenital coronary anomalies
Metabolic toxicity (e.g., carbon monoxide)
Drug-induced infarction (e.g., albuterol)
Pulmonary embolism

**Key Learning Points**

- Non-invasive perfusion imaging such as MCE, radionuclide techniques, dobutamine echocardiography, magnetic resonance, and positron emission tomography have all been shown to have value in diagnosing coronary artery disease.
- MCE is a new echocardiographic modality that relies on microbubbles to scatter ultrasound efficiently and hence improve imaging.
- Because they are confined to the vasculature, contrast agents provide a unique red-cells view of myocardial perfusion.
- By assessing rate of perfusion and myocardial volume, tissue perfusion, and hence myocardial viability, can be accurately quantified.
- An assessment of viability can guide immediate and subsequent treatment strategies in patients with a variety of cardiac conditions, including MI.
- Fast becoming the standard of care in the treatment of AMI, MCE provides prognostic value even for patients undergoing primary angioplasty.
- While indicated for only diagnostic purposes at present, the capacity to load microbubbles with active therapy (drug or gene) and rupture them at will with ultrasound at the site of disease promises that they will be used for directed therapeutic purposes.
- MCE carries a learning curve: the dissemination of MCE must be carried out with specific attention to technical excellence and training to ensure that existing trial results can be adequately translated to real-world patient care.
- When microbubbles are ultimately licensed, ascent of the learning curve, coupled with an appreciation of the myocardial segments and circumstances that may limit the accuracy of the technique, will ensure optimal patient outcomes.

## Abbreviations

AMI: acute myocardial infarction
LAD: left anterior descending
MCE: myocardial contrast echocardiography
MI: myocardial infarction
